# Epidemiological characteristics of foodborne disease outbreaks in Chongqing from 2003 to 2023

**DOI:** 10.1371/journal.pone.0342130

**Published:** 2026-03-11

**Authors:** Jingrong Chen, Qing Liu, Shuquan Luo, Yuan He, Li Cheng, Ping Feng, Fang Li, Wenming Jiang

**Affiliations:** 1 Chongqing Municipal Academy of Preventive Medicine, Chongqing Center for Disease Control and Prevention, Chongqing, China; 2 Chongqing Chemical Industry Vocational College, Chongqing, China; University of Patras, GREECE

## Abstract

Foodborne diseases have a high incidence and widespread occurrence, affecting a significant number of individuals, making them one of the most pressing public health issues globally. The situation regarding foodborne diseases in China is similarly concerning. This study collected data on foodborne disease outbreaks reported by 39 districts and counties in Chongqing from 2003 to 2023 and analyzed the epidemiological characteristics. The aim was to understand the patterns and trends of foodborne disease outbreaks in Chongqing, thereby providing a scientific basis for developing effective prevention and control strategies. From 2003 to 2023, a total of 767 foodborne disease outbreaks were reported in Chongqing, resulting in 10,462 individual cases and 43 fatalities. Domestic settings were identified as the primary location for these incidents, accounting for 46.4% (356/767) of the total occurrences, followed by hotel and restaurant, which contributed 25.0% (192/767). The majority of incidents occurred between May and October. The highest number of incidents was associated with the consumption of poisonous mushrooms, totaling 204 cases, which represents 26.6% (204/767) of all reported incidents. This was followed by meat and meat products, which accounted for 94 incidents, or 12.3% (94/767). Poisonous mushrooms and aconitines emerged as the leading pathogenic factors contributing to fatalities, responsible for 27.9% (12/43) and 16.3% (7/43) of the total deaths, respectively. In recent years, the epidemiological characteristics of foodborne disease outbreaks in the region have evolved. Since 2020, surveillance datum has indicated that mushroom-related events have emerged as the predominant cause of both foodborne disease incidents and associated fatalities in Chongqing. It is essential to enhance monitoring of cases and incidents, improve incident response and early warning mechanisms, conduct comprehensive analyses of the factors contributing to the high incidence of these events, and implement targeted supervision and education for critical locations, pathogenic factors, and personnel.

## Introduction

Foodborne diseases refer to diseases caused by pathogenic agents, including toxic and harmful substances that enter the human body through ingestion. The attack rate of these diseases ranks among the highest of all disease categories. However, what is currently known represents only the tip of the iceberg. It is estimated that the rate of unreported cases in developed countries is as high as 90%, while in developing countries, it exceeds 95% [1]. According to the World Health Organization (WHO) epidemiological reference group on the burden of foodborne diseases (FERG 2007–2015), in 2010, foodborne diseases affected 600 million individuals, resulted in 420,000 deaths, and led to 33 million cases of disability, imposing a significant burden on public health and the economy [2]. However, the underreporting of foodborne diseases remains a significant issue. Currently, the cases reported through monitoring represent only the tip of the iceberg [3]. Notably, 30% of those affected by foodborne diseases are children under the age of five, who represent 9% of the global population [4]. Due to their high incidence, widespread nature, and the large number of individuals affected, foodborne diseases have emerged as one of the most pressing public health challenges worldwide [5]. The situation in China is similarly concerning. From 2003 to 2017, a total of 19,517 outbreaks of foodborne diseases were reported, resulting in 235,754 cases of illness, 107,470 hospitalizations, and 1,457 deaths [6].

Foodborne disease outbreaks refer to occurrences of food-related illnesses in which two or more cases exhibiting similar clinical manifestations have been confirmed through epidemiological investigation [7]. China has established criteria and guidelines for the assessment and management of foodborne disease outbreaks associated with various pathogens. For instance, the Ministry of Health introduced a health industry standard in 1996. In 2023, the Technical Guide for the Identification and Management of Foodborne Diseases was revised and published [8].

In recent years, China has made significant advancements in its foodborne disease monitoring system. Since 2000, the country has established a “two-network” monitoring framework to address food contamination and foodborne diseases. “Two Networks” is the core system in China designed to enhance the capacity for monitoring, early warning, and traceability of foodborne diseases. Its goal is to achieve “early detection, early warning, and early control” of food safety hazards. This system comprises two components: the food pollutant monitoring network and the foodborne disease monitoring network [9]. Following the implementation of the Food Safety Law in 2009, China initiated the development of a food safety risk monitoring system. In 2011, the National Food Safety Risk Assessment Center (CFSA) launched both the foodborne disease monitoring system and the foodborne disease outbreak reporting system [10]. The monitoring activities encompass the surveillance of infectious and toxic cases linked to food, the tracking of suspected foodborne aggregation events, and the monitoring of foodborne disease outbreaks. Concurrently, a molecular traceability network utilizing PFGE molecular typing technology was established to monitor positive samples from cases and outbreaks, thereby facilitating the identification of foodborne diseases and events, as well as improving the traceability of pathogenic food sources [11].

Chongqing is located in the southwest region of China, with a permanent population of 32,133,400 as of the end of 2023 [12]. The city is situated in a mid-latitude subtropical climate zone, characterized by a warm, humid, and rainy climate. The occurrence of foodborne disease outbreaks in this area exhibits unique epidemiological characteristics. In 2003, the government promulgated the Emergency Regulations for Public Health Emergencies [13], addressing issues such as inaccurate information, slow response times, and insufficient emergency preparedness. The law mandates that institutions at all levels implement an emergency reporting system that complies with established standards for reporting major foodborne disease outbreaks. In the same year, the Ministry of Health issued the Food Safety Action Plan, which transformed the monitoring of the “two networks” from a major scientific and technological project into a government initiative aimed at ensuring food safety. This initiative was subsequently expanded to 22 provinces and cities, with Chongqing beginning to monitor foodborne disease outbreaks [14]. This paper analyzes the epidemiological features of foodborne disease outbreaks reported in Chongqing from 2003 to 2023. The aim is to understand the current situation and trends in these incidents, thereby providing a scientific basis for the formulation of effective prevention and control strategies for foodborne diseases.

## Materials and methods

### Materials

The data on foodborne disease outbreaks verified by epidemiological investigations in 39 districts and counties of Chongqing were collected through the national foodborne disease monitoring and reporting system. The report includes information on the monitoring area, occurrence time, outbreak site, source of the causal food, number of people exposed, number of cases, number of hospitalizations, number of deaths, causal food, contamination links, and pathogenic factors. The inclusion criteria for the event are as follows: (1) it occurred between 2003 and 2023; (2) clear epidemiological investigation reports were prepared by disease prevention and control institutions at or above the county level; and (3) it was confirmed as an outbreak of foodborne disease. The exclusion criteria are: (1) outbreaks caused by non-foodborne routes; and (2) data that are severely incomplete, making it impossible to determine the three-dimensional distribution of basic epidemiological information. To ensure data quality, all data reported to the monitoring system are reviewed by researchers at the Chongqing Center for Disease Control and Prevention and cross-checked against the original epidemiological investigation reports. If inconsistencies or uncertainties are identified, verification and correction are conducted by consulting the original reports or contacting the relevant reporting units. Records with missing key variables are excluded from the final analysis. A total of 767 cases of foodborne disease outbreaks were reported in these 39 districts and counties of Chongqing from 2003 to 2023.

### Data analysis methods

Excel 2019 was used for data sorting, and OriginPro 2024 was employed for graphing. In R software, the nonparametric Mann-Kendall trend test and linear regression model were used to verify the long-term trends in the number of events, patients, and deaths.

Based on the data of foodborne disease outbreaks in Chongqing from 2003 to 2023, the spatial distribution map of foodborne disease outbreaks in Chongqing was made by using QGIS 3.34.14 software [15]. GeoDa version 1.14.0.0 software was used to perform spatial autocorrelation analysis [16], with the number of foodborne disease events analyzed at the district (county) level as the research units.

## Results

### Basic information

**Fig 1:**
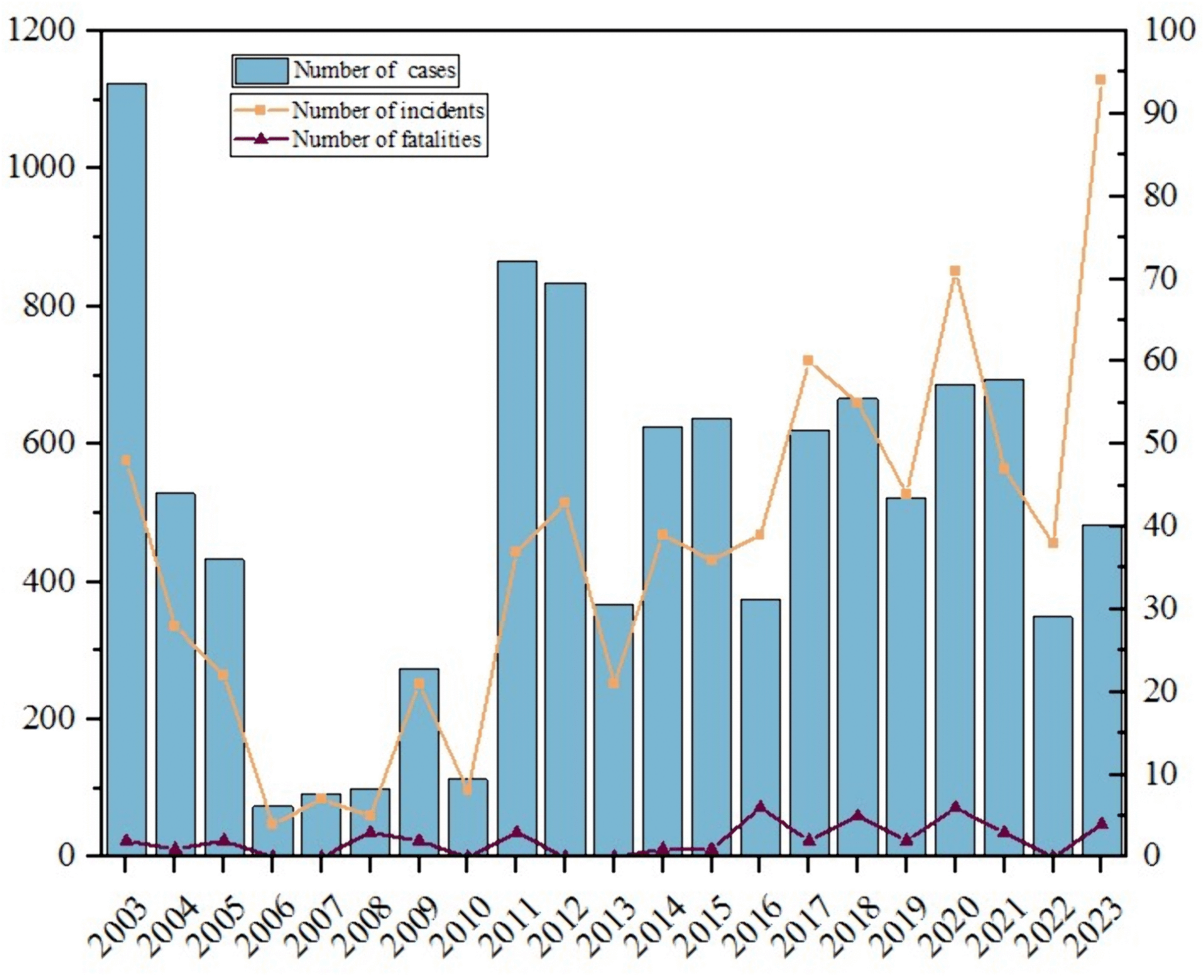
Number of cases, incidents and fatalities of foodborne disease outbreaks.

From 2003 to 2023, a total of 767 foodborne disease outbreaks were reported in Chongqing, affecting 101,114 individuals. Among these, there were 10,462 cases, resulting in an attack rate of 10.4%, and 43 fatalities, corresponding to a mortality rate of 0.4% (S1 Table). On average, approximately 38 cases were reported annually, with no consistent trend in the number of incidents. Between 2006 and 2010, the number of reported incidents decreased significantly; however, starting in 2011, the number of incidents began to rise, with a notable increase observed after 2017. The highest number of reported incidents occurred in 2023, while the peak number of cases was recorded in 2003. The mortality rate was highest in 2008 (Fig 1). The majority of incidents were concentrated in the central city, which accounted for 33.9% (260 out of 767) of the total incidents, and the number of cases represented 39.2% (4,101 out of 10,462) of the total cases. The regions with the highest mortality rates were northeast Chongqing and west Chongqing, accounting for 30.2% (13 out of 43) and 23.3% (10 out of 43) of the total fatalities, respectively. Further analysis revealed a significant upward trend in the number of incident reports (Mann-Kendall τ = 0.498, p = 0.002; linear regression β = 2.51, p < 0.001). However, the number of cases (p = 0.291) and deaths (p = 0.114) did not show significant changes. Global autocorrelation analysis indicated that the 767 events in Chongqing from 2003 to 2023 were randomly distributed across the study area (global Moran’s I = −0.07, Z = −0.43, p = 0.35). Local autocorrelation results identified low-high concentration areas in Jiangbei District, Yuzhong District, and Banan District, while a high-low concentration area was observed in Pengshui Miao and Tujia Autonomous County.

### Distribution of occurrence places

Domestic settings are the primary locations for incidents, accounting for 46.4% (356 out of 767) of the total number of incidents. This is followed by hotels and restaurants, which account for 25.0% (192 out of 767) of the total incidents. An analysis of the incident data reported each year indicates that domestic settings consistently serve as the main sites for foodborne disease outbreaks. The majority of patients are concentrated in hotels (40.2%, 4,201 out of 10,462), domestic settings (21.7%, 2,269 out of 10,462), and school canteens (10.7%, 1,116 out of 10,462). Fatalities primarily occurred in domestic settings (76.7%, 33 out of 43), hotels (11.6%, 5 out of 43), and rural banquets (11.6%, 5 out of 43) (Table 1).

**Table 1:** Distribution of foodborne disease outbreaks in Chongqing from 2003 to 2023.

Place type	Number of events (%)	Number of patients (%)	Number of deaths (%)
Domestic settings	356(46.4)	2269(21.7)	33(76.7)
Catering service venues			
Hotel and restaurant	192(25.0)	4201(40.2)	5(11.6)
Unit canteen	43(5.6)	700(6.7)	0(0.0)
School canteen	38(5.0)	1116(10.7)	0(0.0)
Rural banquet	26(3.4)	503(4.8)	5(11.6)
Food store	23(3.0)	209(2.0)	0(0.0)
Street stalls	17(2.2)	133(1.3)	0(0.0)
Dining delivery unit	11(1.4)	289(2.8)	0(0.0)
Construction site Canteen	3(0.4)	37(0.4)	0(0.0)
School	10(1.3)	392(3.8)	0(0.0)
Others	48(6.3)	613(5.9)	0(0.0)
Total	767(100.0)	10462(100.0)	43(100.0)

### Temporal distribution

**Fig 2:**
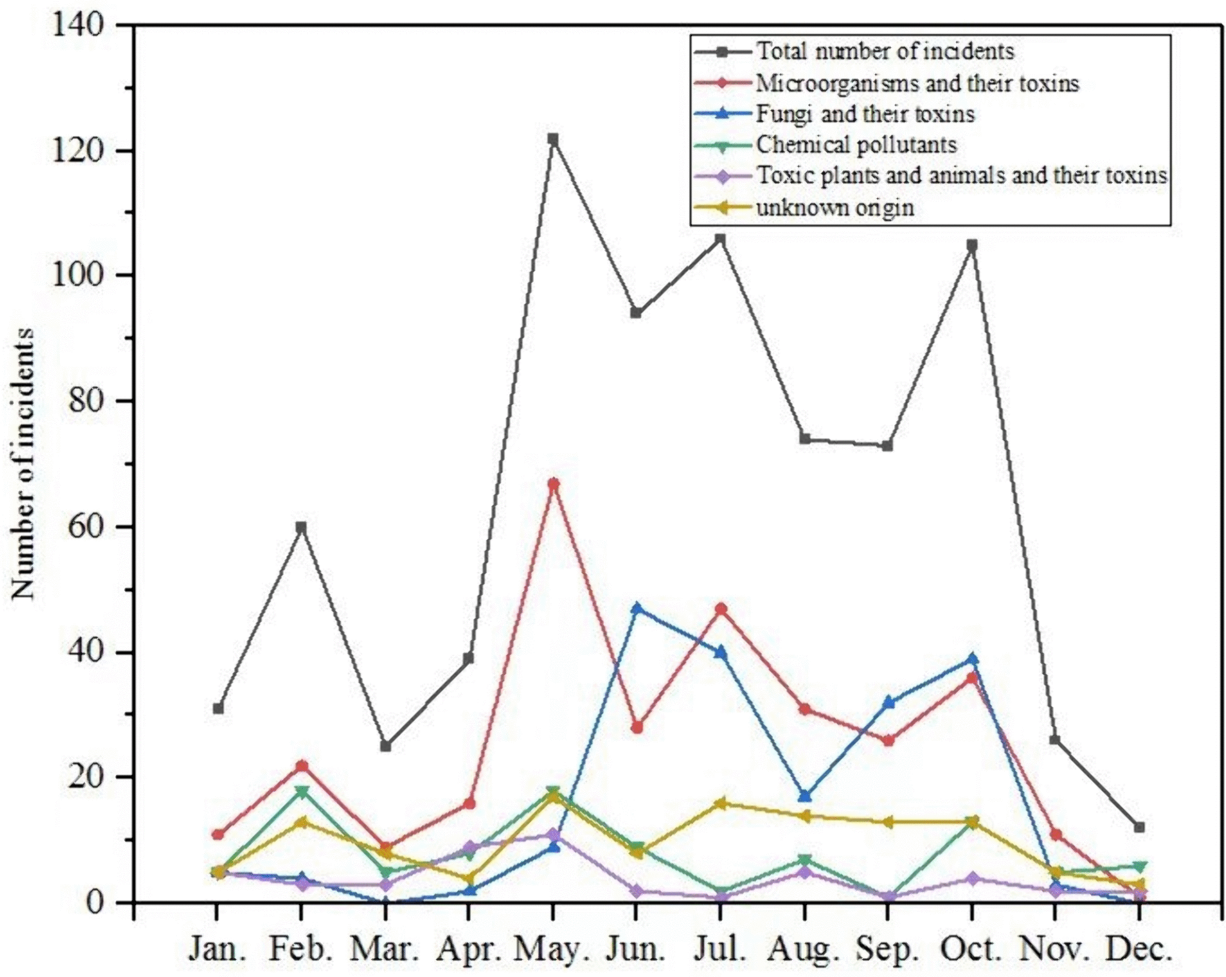
Distribution of outbreaks of foodborne diseases in each month.

From 2003 to 2023, foodborne disease outbreaks in Chongqing predominantly occurred between May and October, with 574 incidents reported, accounting for 74.8% (574/767) of the total number of incidents. These incidents resulted in 7,363 cases, representing 70.4% (7,363/10,462) of the total cases, and led to 28 fatalities. The seasonal characteristics of foodborne disease outbreaks are pronounced, with a higher attack rate observed during the summer and autumn months. The seasonal trends of microorganisms, their toxins, and unexplained outbreaks align closely with those of general events. Fungi and their toxins, particularly poisonous mushrooms, exhibit a seasonal variation, peaking from June to October. In contrast, the seasonal trend of outbreaks caused by other pathogenic factors is less pronounced (Fig 2).

### Distribution of pathogenic factors

From 2003 to 2023, out of 767 reported incidents, 653 were identified, accounting for 85.1% of the total. Incidents caused by pathogenic microorganisms and their toxins, fungi and their toxins, chemical pollutants, poisonous plants and their toxins, and poisonous animals and their toxins represented 39.8% (305/767), 26.6% (204/767), 12.7% (97/767), 5.2% (40/767), and 0.9% (7/767), respectively. Poisonous mushrooms, aconitine, and methanol were the primary pathogenic factors responsible for fatalities, accounting for 27.9% (12/43), 16.3% (7/43), and 11.6% (6/767), respectively. Analyzing the trend of these incidents, it is evident that prior to 2020, foodborne diseases in Chongqing were predominantly caused by pathogenic microorganisms and their toxins, followed by poisonous mushrooms.

**Fig 3:**
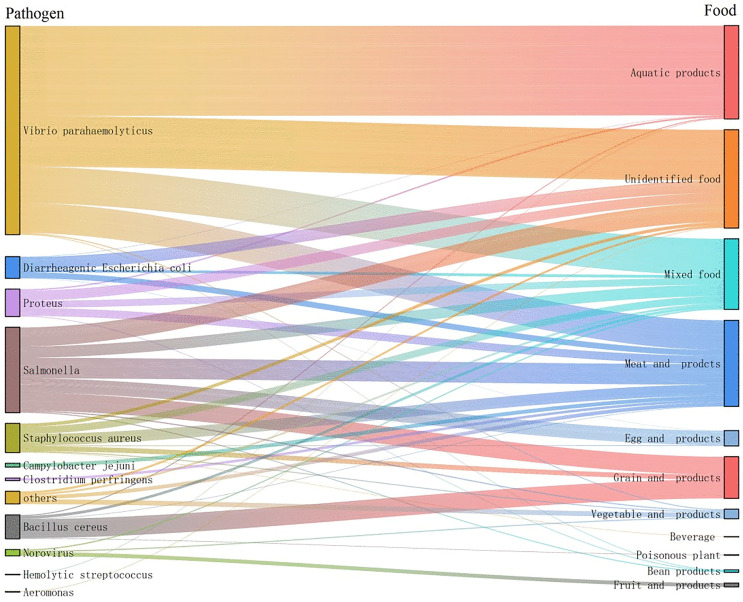
Distribution of pathogenic factor-food combination of microbial foodborne disease outbreaks.

Among pathogenic microorganisms, *Vibrio parahaemolyticus*, *Salmonella*, and *Staphylococcus aureus* are the three most significant. The primary pathogenic factors and associated food combinations are as follows: *Vibrio parahaemolyticus* with aquatic products, *Salmonella* with meat and meat products, and *Staphylococcus aureus* along with its toxins, also associated with meat and meat products (Fig 3).

## Discussion

This study revealed a unique epidemiological model by analyzing outbreak data of foodborne diseases in Chongqing from 2003 to 2023. The main findings can be summarized as follows: the number of reported incidents showed a significant upward trend; households were the primary outbreak sites; incidence exhibited a clear peak during summer and autumn; and the profile of pathogenic factors changed significantly. The seasonal peak in incidence aligns closely with the nationwide pattern of occurrence [17].

The phenomenon of low-high concentration areas may be related to several factors. First, the aforementioned areas, as the core urban regions of the city, are home to large-scale catering establishments, government canteens, and schools. These institutions typically have relatively standardized management practices, a strong capacity to control food safety risks, and consequently, fewer actual outbreaks. Second, this phenomenon may be influenced by the availability of medical resources and the effectiveness of foodborne disease reporting systems. High-low concentration areas may reflect the presence of specific local risk factors, such as frequent communal dinners in rural regions, limited food storage facilities, unsafe drinking water, or local food source hazards like toxic plants, all of which can contribute to an increase in local outbreaks. Additionally, in recent years, improvements in grassroots monitoring sensitivity and heightened awareness of reporting have likely led to more comprehensive identification and reporting of incidents.

One of the most notable findings of this study is the shift in the predominant pathogen profile. Since 2020, incidents involving poisonous mushrooms have surpassed those caused by microbial pathogens, becoming the leading type of outbreak and cause of death. This trend may be attributed to Chongqing’s warm and humid climate, which favors mushroom growth, as well as the traditional practice among residents of foraging and consuming wild mushrooms. Notably, although Chongqing is an inland region, *Vibrio parahaemolyticus* has emerged as the most significant microbial pathogen, contrasting with the common understanding that *Vibrio parahaemolyticus* typically predominates in coastal areas, while *Salmonella* accounts for the majority of outbreaks in inland regions [18]. This finding suggests that aquatic products constitute a notable portion of the diet in inland areas, warranting special attention in prevention and control strategies. The study also found that households were the primary setting for outbreaks, consistent with most global reports [19,20]. However, the proportion of family outbreaks in China (approximately 59.96%) [21] is significantly higher than the estimate reported by the European Union (39.1%) [22]. This discrepancy may be related not only to differences in food culture and preparation habits but also to variations in the sensitivity of monitoring systems in detecting family outbreaks. The EU report explicitly acknowledges that its family outbreak data may be underestimated [22]. This suggests that China’s monitoring system may be more sensitive in capturing family outbreaks, but it may also indicate more severe underreporting of outbreaks in other settings, such as catering services.

This study found that the number of reported foodborne disease incidents in China exhibited a significant upward trend from 2003 to 2023, while the number of cases and deaths did not show a statistically significant long-term change. This pattern suggests that the increase in reported incidents may primarily result from the continuous enhancement of monitoring and reporting capabilities, rather than an actual rise in the disease burden. Notably, the upward trend in incident reports closely aligns with key developmental stages of China’s food safety monitoring system. Although the number of incidents increased significantly over time, the number of cases and deaths remained relatively stable during the same period. This trend likely reflects improved monitoring sensitivity rather than a deterioration in the actual risk of foodborne diseases. Future research should incorporate population-standardized attack rates, mortality rates, or sub-cause data to more accurately assess changes in the true disease burden.

The high mortality rate from poisonous mushroom poisoning in China [23,24] is significantly higher than the global average [25], posing a serious public health threat. Consequently, it is considered one of the deadliest foodborne public health emergencies in China [26], with notable regional disparities, particularly in southwestern China [10,27,28]. Additionally, defining the primary pathogenic factor–food combinations—such as *Vibrio parahaemolyticus* with aquatic products, *Salmonella* with meat and meat products, and *Staphylococcus aureus* and its toxins with meat and meat products—provides a scientific basis for developing targeted intervention measures along high-risk food chains.

This study has several limitations. First, the data were obtained from a passive monitoring system, which is influenced by the reporting awareness of medical institutions, diagnostic capabilities, and patients’ treatment-seeking behavior. Consequently, underreporting may occur, particularly in cases with mild symptoms or in grassroots areas. Second, between 2003 and 2023, the monitoring system underwent multiple adjustments, and the early case definitions and detection capabilities were limited, affecting the comparability of long-term trends. Third, a significant proportion of incidents lack etiological confirmation, restricting in-depth analysis of pathogenic factors. Additionally, exposure information is incomplete, making detailed risk factor modeling difficult. Finally, the data primarily originate from the medical and health system and do not integrate multi-sectoral information, such as market supervision data, which may lead to an underestimation of the overall risk.

## Conclusion

As a significant public health issue globally, foodborne diseases are exhibiting an alarming trend in incidence in China. This study collected data on foodborne disease outbreaks reported by 39 districts and counties in Chongqing from 2003 to 2023 and conducted a systematic epidemiological analysis to provide a scientific basis for the formulation of prevention and control strategies. During the study period, 767 foodborne disease outbreaks were reported in Chongqing, involving 10,462 individuals and resulting in 43 deaths. Domestic settings were identified as the primary site of incidents, accounting for 46.4%, followed by hotels and restaurants. The peak incidence occurred between May and October, with poisonous mushrooms being the leading cause of incidents and fatalities since 2020, followed by meat and meat products. Over time, poisonous mushrooms have increasingly supplanted microbial pathogens as the primary cause of illness and death. Accidental consumption within domestic settings is a significant contributing factor, with *Vibrio parahaemolyticus* identified as the main microbial pathogen. Based on the research findings, it is recommended to enhance information monitoring of foodborne disease cases in medical institutions and establish a robust risk early warning mechanism. Additionally, it is essential to conduct public awareness research on food safety risks and implement effective health communication strategies. Furthermore, a systematic inventory of toxic plants and mushrooms should be compiled, a poisoning case database established, high-risk factors identified through scientific research, and targeted prevention and control measures implemented to improve the efficiency of foodborne disease prevention and control.

## Supporting information

S1 Table Distribution of pathogenic factors of events. (DOCX)

## References

[1] Dudeja P, Gupta RK, Minhas AS. Food safety in the 21st century: Public health perspective. Academic Press; 2016.

[2] WHO. WHO estimates of the global burden of foodborne diseases. 2020. [Cited 2024 January 2]. http://www.unscn.org/www.unscn.org/en/news-events/recent-news?idnews=2102

[3] Chen J. Food safety-A major public health issue in China. Chinese Journal of Epidemiology. 2003;24(8):649–50. 15481527

[4] WHO. WHO’s first ever global estimates of foodborne diseases find children under 5 account for almost one third of deaths [EB/OL]. (2015-12-03). [2022-07-21]. http://www.who.int./mew/item/03-12-2015-who-s-first-ever-global-estimates-of-foodborne-diseases-find-children-under-5--account-for-almost-one-third-of-deaths

[5] Havelaar AH, Kirk MD, Torgerson PR, et al. World Health Organization global estimates and regional comparisons of the Burden of Foodborne Disease in 2010. PLoS Med. 2015;12(12):e1001923. 10.1371/journal.pmed.1001923 PMC4668832 26633896

[6] Li WW, Pires SM, Liu Z. Surveillance of foodborne disease outbreaks in China, 2003–2017. Food Control. 2020;118:107359. 10.1016/j.foodcont.2017.08.010 PMC7125948 32288325

[7] National Health Commission of the People’s Republic of China. Technical Guidelines for Foodborne Disease Surveillance and Reporting (Trial). 2019. [Cited 2025 June 25]. https://www.nhc.gov.cn/wjw/c100175/201910/e6d5b1d1dada495ab1730108db4e40ed.shtml

[8] National Health Commission of the People’s Republic of China. Technical Guidelines for the Diagnosis and Management of Foodborne Diseases. 2019. [Cited 2023 August 17]. https://www.gov.cn/zhengce/zhengceku/2019-11/13/content_5451649.htm

[9] Wu YN. Overview of data requirements for food safety risk monitoring in China and abroad. Chinese Journal of Food Hygiene. 2011;23(1):8–12.

[10] Liu J, Bai L, Li W, Han H, Fu P, Ma X, et al. Trends of foodborne diseases in China: lessons from laboratory-based surveillance since 2011. Front Med. 2018;12(1):48–57. doi: 10.1007/s11684-017-0608-6 29282610

[11] Wu Y-N, Liu X-M, Chen Q, Liu H, Dai Y, Zhou Y-J, et al. Surveillance for foodborne disease outbreaks in China, 2003 to 2008. Food Control. 2018;84:382–8. doi: 10.1016/j.foodcont.2017.08.010 32288325 PMC7125948

[12] Chongqing Provincial Bureau of Statistics. https://tjj.cq.gov.cn/

[13] Nie Y, Yin C, Tang XC. Comparative analysis of food poisoning and emergency countermeasures in China from 1985 to 2011. Food Science. 2013;34(5):218–22.

[14] Wang MQ, Liu XM, Wang ZT. Studies on National Surveillance System for Food Contaminations and Foodborne Diseases in China. Chinese Journal of Food Hygiene. 2006;18(6):491–7.

[15] Shaira H, Naik PR, Pracheth R, Nirgude AS, Nandy S, Hiba MM, et al. Epidemiological profile and mapping geographical distribution of road traffic accidents reported to a tertiary care hospital, Mangaluru using quantum geographic information system (QGIS). J Family Med Prim Care. 2020;9(7):3652–6. doi: 10.4103/jfmpc.jfmpc_190_20 33102345 PMC7567286

[16] Liu Q, Liu L, Zhang T. Epidemiological characteristics and spatiotemporal clustering of hand foot and mouth disease in Dali, Yunnan, 2016-2021. Disease Surveillance. 2023;38(1):75–81.

[17] Cheng H, Zhao J, Zhang J. Attribution analysis of household foodborne disease outbreaks in China, 2010-2020. Foodborne Pathog Dis. 2023;20(8):358–67. 37506344 10.1089/fpd.2022.0070

[18] Wang S, Duan H, Zhang W, Li J-W. Analysis of bacterial foodborne disease outbreaks in China between 1994 and 2005. FEMS Immunol Med Microbiol. 2007;51(1):8–13. doi: 10.1111/j.1574-695X.2007.00305.x 17666075

[19] Schlinkmann KM, Razum O, Werber D. Characteristics of foodborne outbreaks in which use of analytical epidemiological studies contributed to identification of suspected vehicles, European Union, 2007 to 2011. Epidemiol Infect. 2017;145(6):1231–8. doi: 10.1017/S0950268816003344 28162104 PMC9507843

[20] Langiano E, Ferrara M, Lanni L, Viscardi V, Abbatecola AM, De Vito E. Food safety at home: knowledge and practices of consumers. Z Gesundh Wiss. 2012;20(1):47–57. doi: 10.1007/s10389-011-0437-z 22347771 PMC3268974

[21] Li HQ, Guo YC, Liu ZT, et al. Analysis of foodborne disease outbreaks in China’s Mainland in 2022. Chinese Journal of Food Hygiene. 2024;36(08):962–7.

[22] European Food Safety Authority, European Centre for Disease Prevention and Control. The European Union One Health 2020 Zoonoses Report. EFSA J. 2021;19(12):e06971. 10.2903/j.efsa.2021.6971 PMC9624447 36329690

[23] Chan CK, Lam HC, Chiu SW, Tse ML, Lau FL. Mushroom poisoning in Hong Kong: a ten-year review. Hong Kong Med J. 2016;22(2):124–30. doi: 10.12809/hkmj154706 26980450

[24] He Q, Xie L, Ma P. Analysis of current situation of poisoning caused by poisonous animals, poisinous plants, and poisonous mushrooms in China. Adverse Drug Reactions Journal. 2013;15(1):6–10.

[25] Zhou J, Yuan Y, Lang L. Analysis of hazard in mushroom poisoning incidents in china mainland. Chin J Emerg MED. 2016;25(6):724–8.

[26] Li WW, Pires SM, Liu ZT. Mushroom poisoning outbreaks-China, 2010-2020. China CDC Wkly. 2021;3(24):518–22. 34594925 10.46234/ccdcw2021.134 PMC8393043

[27] Li JY, Guo YC, Li WW. Analysis of foodborne disease outbreaks within households from 2002-2016. Modern Prev Med. 2018;45(8):1499–503.

[28] Li Y, Huang Y, Yang J, Liu Z, Li Y, Yao X, et al. Bacteria and poisonous plants were the primary causative hazards of foodborne disease outbreak: a seven-year survey from Guangxi, South China. BMC Public Health. 2018;18(1):519. doi: 10.1186/s12889-018-5429-2 29669556 PMC5907191

