# Supplementary material for: Epidemiological characteristics of foodborne disease outbreaks in Chongqing from 2003 to 2023
Source: PLoS One. 2026 Mar 11;21(3):e0342130. doi: 10.1371/journal.pone.0342130 (PMC12978440; doi:10.1371/journal.pone.0342130)
Supplement: S1 Table — (DOCX) [file pone.0342130.s001.docx]

Table S1 Distribution of pathogenic factors of events

| Pathogenic factor | | Number of events (%) | Number of patients (%) | Death toll (%) |
| --- | --- | --- | --- | --- |
| Pathogenic microorganisms and their toxins | *Vibrio Parahaemolyticus* | 104(13.56) | 3324(31.77) | 0(0.00) |
|  | *Salmonella* | 85(11.08) | 1360(13.00) | 0(0.00) |
|  | *Staphylococcus aureus* and its toxin | 35(4.56) | 461(4.41) | 0(0.00) |
|  | *Escherichia coli* | 23(3.00) | 343(3.28) | 0(0.00) |
|  | *Proteus* | 20(2.61) | 438(4.19) | 0(0.00) |
|  | *Bacillus cereus* | 14(1.83) | 378(3.61) | 0(0.00) |
|  | *Clostridium perfringens* | 3(0.39) | 41(0.39) | 0(0.00) |
|  | *Norovirus* | 3(0.39) | 102(0.97) | 0(0.00) |
|  | *Campylobacter jejuni* | 1(0.13) | 62(0.59) | 0(0.00) |
|  | *Aeromonas* | 1(0.13) | 7(0.07) | 0(0.00) |
|  | *Streptococcus hemolyticus* | 1(0.13) | 8(0.08) | 0(0.00) |
|  | Mixed factor | 2(0.26) | 23(0.22) | 0(0.00) |
|  | Others | 13(1.69) | 179(1.71) | 0(0.00) |
| Fungi and their toxins | Poisonous mushroom | 204(26.60) | 863(8.25) | 12(27.91) |
| Chemical pollutant | Food additives | 57(7.43) | 329(3.14) | 10(23.26) |
|  | Pesticide residue | 13(1.69) | 61(0.58) | 0(0.00) |
|  | N- nitroso compound | 7(0.91) | 80(0.76) | 0(0.00) |
|  | Tetramine | 7(0.91) | 47(0.45) | 1(2.33) |
|  | Methyl alcohol | 3(0.39) | 47(0.45) | 5(11.63) |
|  | Mixed factor | 1(0.13) | 7(0.07) | 0(0.00) |
|  | Others | 9(1.17) | 57(0.54) | 2(4.65) |
| Toxic animals and their toxins | Bufotoxin | 4(0.52) | 15(0.14) | 1(2.33) |
|  | Animal thyroid gland | 1(0.13) | 3(0.03) | 0(0.00) |
|  | Bee pupa (bee venom) | 1(0.13) | 6(0.06) | 2(4.65) |
|  | Others | 1(0.13) | 2(0.02) | 1(2.33) |
| Poisonous plants and their toxins | Saponins and hemagglutinin (beans, etc.) | 8(1.04) | 143(1.37) | 0(0.00) |
|  | Aconitine (Radix Aconiti Kusnezoffii, Radix Aconiti Lateralis, etc.) | 6(0.78) | 35(0.33) | 7(16.28) |
|  | Scopolamine (Datura stramonium, Belladonna, etc.) | 4(0.52) | 18(0.17) | 0(0.00) |
|  | Masangguo | 3(0.39) | 8(0.08) | 0(0.00) |
|  | Tung oil (Jatropha acid and Isojatropha acid) | 2(0.26) | 76(0.73) | 0(0.00) |
|  | Poisonous wild vegetables | 2(0.26) | 11(0.11) | 0(0.00) |
|  | Phytotoxin others | 15(1.96) | 424(4.05) | 0(0.00) |
| Unknown factor |  | 114(14.86) | 1504(14.38) | 2(4.65) |
| Total |  | 767(100.00) | 10462(100.00) | 43(100.00) |
